# The effectiveness of gentamicin in the treatment of *Neisseria gonorrhoeae*: a systematic review

**DOI:** 10.1186/2046-4053-3-104

**Published:** 2014-09-19

**Authors:** Emma Hathorn, Divya Dhasmana, Lelia Duley, Jonathan DC Ross

**Affiliations:** 1 Whittall Street Clinic, University Hospitals Birmingham, Birmingham B6 4DH, UK; 2 Nottingham Clinical Trials Unit, Nottingham Health Science Partners, Queen’s Medical Centre, Nottingham, UK

**Keywords:** Gonorrhoea, *Neisseria gonorrhoeae*, Gentamicin, Treatment

## Abstract

**Background:**

A high level of resistance in *Neisseria gonorrhoeae* has developed against penicillins, sulphonamides, tetracyclines and quinolones, and recent surveillance data have shown a gradual reduction in sensitivity to current first-line agents with an upward drift in the minimum inhibitory concentration of ceftriaxone. Laboratory sensitivity testing suggests that gentamicin, an aminoglycoside, may be an effective treatment option for gonorrhoea infection when used as a single intramuscular dose.

**Methods:**

A search of electronic reference databases and grey literature was used to identify randomised trials and well-conducted prospective studies with concurrent controls evaluating single-dose gentamicin against placebo or a comparator regimen in the treatment of uncomplicated gonorrhoea infection in men and women aged 16 years and over. The primary outcome was microbiological cure of *N. gonorrhoeae*.

**Results:**

Eight hundred and thirty-nine studies were identified, of which five (1,063 total participants) were included. All five studies administered single-dose gentamicin via intramuscular injection to men with uncomplicated gonococcal urethritis. Three studies were randomised trials, one was quasi-randomised and one was non-randomised but included a comparator arm. Comparator antibiotics included an alternative aminoglycoside or antibiotic used in the syndromic management of male urethritis. Methodology was poorly described in all five included studies. The high risk of bias within studies and clinical heterogeneity between studies meant that it was inappropriate to pool data for meta-analysis. Cure rates of 62% to 98% were reported with gentamicin treatment. The relative risk of cure was comparable between gentamicin and comparator antibiotics.

**Conclusions:**

The studies identified provide insufficient data to support or refute the efficacy and safety of single-dose intramuscular gentamicin in the treatment of uncomplicated gonorrhoea infection. Additional randomised trials to evaluate gentamicin for this indication are therefore required.

**Systematic review registration:**

PROSPERO CRD42012002490

## Background

Gonorrhoea, caused by *Neisseria gonorrhoeae*, is the second most common bacterial sexually transmitted infection in the UK. The number of gonorrhoea diagnoses continues to rise with latest data indicating a 52% increase in England from 16,835 to 25,525 infections between 2010 and 2012 [1]. The highest rates of infection are found in residents of urban areas, and infection is concentrated in core groups such as young people and men who have sex with men (MSM). Of male diagnoses in 2012, 42% were reported in MSM and 55% of heterosexual diagnoses were in those aged 15 to 24 years [1]. A number of factors, in addition to continuing levels of unsafe sexual behaviour, may have contributed to the observed increase in diagnosis of gonorrhoea including the introduction of highly sensitive nucleic acid amplification tests (NAATs), the introduction of self-testing including extra-genital sites (pharyngeal and rectal sites in MSM), an increase in sexual health screening following the roll-out of the National Chlamydia Screening Programme, and improvements in reporting and surveillance. However, the rise in incident infection reported in the UK mirrors the global trend; the World Health Organization (WHO) estimates gonorrhoea to represent 106.1 million of the 498.9 million new cases of curable sexually transmitted infections (syphilis, chlamydia, gonorrhoea and trichomoniasis) worldwide [2].

*N. gonorrhoeae* are intracellular gram-negative bacteria transmitted via sexual contact. They primarily infect the mucous membranes of the urethra, endocervix, rectum, pharynx and conjunctiva. Infection of the genital tract causes local inflammation that can result in dysuria, discharge, genital discomfort and pain. Infection in women may spread to the fallopian tubes and ovaries causing pelvic inflammatory disease (PID). Complications include infertility, chronic pelvic pain and ectopic pregnancy and can result in considerable physical and emotional morbidity in addition to a significant financial burden on health-care services [3,4]. An estimate of the average lifetime costs for women who develop complications is $6,350 for chronic pelvic pain, $6,840 for ectopic pregnancy and $1,270 for infertility [4].

Gonorrhoea has consistently been identified as a risk factor for incident HIV infection in both heterosexual and MSM populations [5-7]. This is thought to result from increased HIV viral shedding in genital secretions [8,9] and from an increased concentration of target cells for HIV in the locally inflamed mucosa found in individuals with gonorrhoea [10]. Ensuring effective gonorrhoea testing and treatment is therefore important to both reduce the global incidence of curable sexually transmitted infections and control the spread of HIV.

Testing guidance advocates the use of NAATs to detect gonorrhoea [11-14]. These tests benefit from high sensitivity and specificity, quick turnaround times, ability to use non-invasive specimens from patients and permitting dual screening for chlamydia and gonorrhoea at extra-genital sites. However, to allow antibiotic susceptibility testing, a sample for culture is also required. *In vitro* susceptibility testing is used to guide individual patient management in addition to providing data for surveillance programmes. Antibiotic resistance in *N. gonorrhoeae* is a continuing problem, and surveillance of antibiotic resistance with a change in empirical treatment when resistance occurs in >5% of isolates is recommended [15]. This is of particular importance in resource-limited settings where testing for *N. gonorrhoeae* is difficult and individuals are typically treated using syndrome-based algorithms.

A high level of resistance in *N. gonorrhoeae* has developed against penicillins, sulphonamides, tetracyclines and fluoroquinolones, which are now no longer recommended for use. Current guidelines recommend a single dose of intramuscular ceftriaxone with or without the addition of a single oral dose of azithromycin for the treatment of uncomplicated gonorrhoea infection [12,14,16,17]. The European Centre for Disease Prevention and Control (ECDC) has proposed a working case definition for confirmed treatment failure that includes both clinical and laboratory criteria [18]. The precise resistance breakpoint for cephalosporin antibiotics is unknown, but surveillance data from the gonococcal isolate surveillance projects in both the UK and US have reported an upward drift in the mean inhibitory concentration (MIC) of ceftriaxone (UK: 13% with MIC over 0.03 mg/l in 2010 cf. 1% in 2007 [19]; US: 0.05% with MIC over 0.125 mcg/ml in 2006 cf. 0.5% in 2010 [20]). Clinical failure of cephalosporins has now been reported in Japan and Europe [21-23].

The mechanism of resistance to cephalosporin antibiotics is not fully understood. Plasmid-mediated resistance has not been observed, but a number of chromosomal mechanisms, including the presence of mosaic *penA* genes and mutations in *penA*, *penB*, *mtrR* promoter and *mtrR* genes, have been reported. Mosaicism of the *penA* gene that encodes penicillin-binding protein 2 (PBP2) is thought to be the predominant mechanism causing cephalosporin resistance. Penicillin-binding proteins are involved in the synthesis of peptidoglycan, a major component of bacterial cell walls. Mosaic sequences of PBP2, resulting from recombination events involving *penA* gene sequences from other *Neisseria* species, have been identified in clinical isolates that demonstrate reduced susceptibility to cefixime and ceftriaxone [24-26]. Options to treat gonorrhoea if cephalosporins become ineffective are severely limited.

Gentamicin, an aminoglycoside antibiotic, is known to be clinically effective in the treatment of gram-negative infections, exerting both a bacteriostatic and bactericidal effect. It is an inexpensive antibiotic and has been used successfully to treat genital gonorrhoea infections in resource-limited settings. Studies in Malawi have shown high susceptibility of gonococcal isolates to gentamicin *in vivo* and clinical cure rates of approximately 95% when used in combination with doxycycline [27,28]. Whilst it is not included in current UK or European gonorrhoea treatment guidelines, an evaluation of gentamicin susceptibility of gonorrhoea isolates across 17 European countries was performed in 2009 in response to the emergence of decreased susceptibility to third-generation, extended-spectrum cephalosporins. The majority of MICs for genital and rectal isolates fell within a narrow range: 95% of isolates within 4–8 mg/l and 79% of isolates demonstrating an MIC of 8 mg/l [29]. Cephalosporin-resistant gonococci are unlikely to exhibit cross-resistance to gentamicin given its bacteriostatic action via the bacterial ribosome in contrast to the action of cephalosporins on bacterial cell wall synthesis. Administration of gentamicin is limited to either the intravenous or intramuscular routes, and the optimal dose for uncomplicated genital infections is not known. However, a single intramuscular injection of antibiotic lends itself to outpatient management and may reduce the risk of vestibular and renal toxicity that is seen with extended high trough drug concentrations.

In view of concerns about decreasing sensitivity of *N. gonorrhoeae* to current first-line agents, there is a clear need to identify treatment options. This systematic review assesses the clinical effectiveness and safety of gentamicin for the treatment of *N. gonorrhoeae*.

## Methods

The review protocol was registered with PROSPERO (CRD42012002490) [30].

### Criteria for considering studies for the review

#### Types of study

Randomised controlled trials, quasi-randomised trials and prospective studies with concurrent controls and consistent treatment assignment were eligible for inclusion. Studies with historical controls, before and after studies, case series and case reports were excluded.

#### Types of participants

Studies recruiting men and women aged 16 years or over receiving their first antibiotics as an inpatient or outpatient for a microbiological diagnosis of gonorrhoea at any anatomical site were included. Gonorrhoea was diagnosed by microscopy, culture or nucleic acid amplification tests.

#### Types of intervention

Studies in which gentamicin was given at any single dose intramuscularly or intravenously were eligible for inclusion. To enable assessment of gentamicin efficacy, studies in which gentamicin was given as part of a combined antibiotic regimen were excluded. Comparators included no treatment, placebo or any alternative antibiotic given either orally or parenterally.

#### Types of outcome

##### Primary outcome

The primary outcome is microbiological cure of *N. gonorrhoeae* (negative microscopy, culture or nucleic acid amplification test).

##### Secondary outcomes

The secondary outcomes are the following:

• Clinical resolution of symptoms (dysuria, genital discharge, genital pain or abdominal pain).

• Need for additional antibiotic therapy.

• Adverse events (rash, allergy, injection site discomfort, renal dysfunction, hearing loss, vestibular dysfunction, other reported adverse events attributed to antibiotic).

• Hospital attendance (admission to hospital, unscheduled clinic attendance).

### Search strategy for identification of studies

#### Electronic searches

A search strategy was developed and used to identify relevant studies (Additional file 1). Databases were searched on 11th May 2012 and 27th July 2012 and updated on 2nd June 2014 as follows: MEDLINE from PubMed 1950 to present, Embase 1980 to present, CINAHL 1981 to present, CAB Abstracts, EThOS and the Cochrane Central Register of Controlled Trials. Searches were repeated of http://www.clinicaltrials.gov and http://www.who.int/trialsearch to identify ongoing trials. Papers published in peer-reviewed journals, theses, conference abstracts and reports were included. No language restriction was placed on the search strategy.

#### Searching other resources

Searches were repeated in grey literature to identify any unpublished and ongoing research (Additional file 1). References from included studies were reviewed for further relevant studies.

### Data collection and analysis

#### Study selection

Two reviewers (DD and EH) independently screened titles and abstracts against the inclusion criteria to identify eligible citations. Disagreements were resolved by discussion. Full-text copies were obtained if insufficient information was available and of all studies meeting inclusion criteria.

#### Data extraction

A standardised data extraction form was developed and utilised. Data were independently extracted from the studies, and discrepancies were resolved by discussion and consultation with a third reviewer if necessary.

#### Assessment of risk of bias

Risk of bias in studies was assessed using the risk of bias tool described in the Cochrane Handbook for Systematic Reviews of Interventions [31]. A bias judgement (low risk of bias, high risk of bias or unclear risk of bias) was allocated to each of six domains (sequence generation, allocation of sequence concealment, blinding, incomplete outcome data, selective reporting bias and other bias) within the tool. The tool was developed for assessment of randomised trials, and the Good Research for Comparative Effectiveness (GRACE) checklist, which has been utilised to rate the quality of observational studies of comparative effectiveness, was used to assess non-randomised studies included for data analysis [32].

#### Data synthesis

Characteristics, main findings and risk of bias assessment were tabulated for each study. Levels of attrition were noted for included studies. If data were adequate for meta-analysis, we planned that results be presented as a summary risk ratio with 95% confidence intervals, on an intention-to-treat basis.

Reviewing the studies identified a high level of clinical heterogeneity. The dose of gentamicin varied from 160 to 280 mg, and the primary assessment of cure was based on symptoms, microscopy and culture or was not reported. The definition and assessment of risk of re-infection was not reported in some studies [33-35], and those with potential re-exposure were excluded in one study [36]. Meta-analysis was considered to be inappropriate due to these methodological differences and the results summarised in a tabular format.

## Results

**Figure 1:**
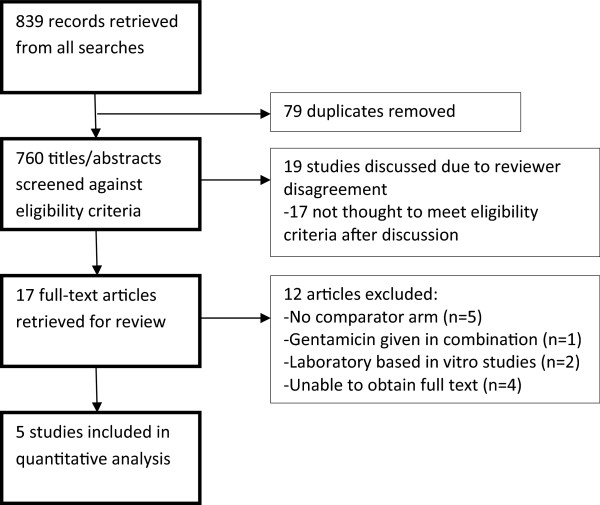
Review profile.

Eight hundred and thirty-nine studies were identified by the search strategy (Figure 1). Two reviewers independently screened all titles and available abstracts. Nineteen studies were discussed due to disagreement, of which 17 were excluded, as they did not meet pre-specified inclusion criteria. Seventeen full-text articles were retrieved and 12 were excluded after further review.

Five studies with a total of 1,063 participants were included in the review: three randomised trials [28,33,34], one quasi-randomised trial [36] and one non-randomised study with a comparator arm [35].

### Description of studies

Table 1 summarises the characteristics of the included studies. All five studies included men with uncomplicated gonococcal urethritis diagnosed by culture [34], identification of gram-negative diplococci on urethral smear [36] or a combination of Gram-stained urethral smear and culture [28,34,36]. Five hundred and twenty-nine men received intramuscular gentamicin in a single dose—160 mg (*n* = 20), 240 mg (*n* = 207) or 280 mg (*n* = 302). No study compared gentamicin to placebo and comparator antibiotics included an alternative aminoglycoside (kanamycin and spectinomycin) or antibiotic used in the syndromic management of male urethritis.

**Table 1:** Characteristics of included studies

**Author**	**Methods**	**Participants**	**Intervention**		**Primary outcome**	**Evaluation of re-infection**
			**Gentamicin**	**Comparator**		
Hira et al. (1984) [36]	Quasi-random (treatment assigned to alternate consecutive patients)	Men with uncomplicated gonorrhoea infection (gram-negative diplococci on urethral smear), Lusaka, Zambia	Single-dose gentamicin 280 mg intramuscular injection (*n* = 302)	Single-dose kanamycin 2 g intramuscular injection (*n* = 113)	Cure	All patients advised to abstain from sexual activity for 2 weeks after therapy.
					Patients in whom *N. gonorrhoea* persisted or re-appeared (as determined by a positive result of a smear or culture) in the absence of sexual activity during the follow-up period were considered to be treatment failure	Patients excluded if reported sexual activity during 2 weeks follow-up period with or without persistent or re-appearing gonorrhoea on culture
Iskandar et al. (1978) [33]	RCT (randomly allocated to 3 groups of 30 patients)	Men with acute gonorrhoea infection (gonorrhoea on Gram stain of urethral smears), Egypt	Single-dose gentamicin 240 mg intramuscular injection (*n* = 30)	Co-trimoxazole (Bactrim, Roche) 8 tablets daily divided into 2 doses for 2 days (*n* = 30). Trimethoprim-sulphametrol (Lidaprim, Ciba) 8 tablets divided into 2 doses for 2 days (*n* = 30)	Cure	One case of re-infection reported in which there was a history of re-exposure.
					Cases with negative smears plus resolution of discharge on day 7 were considered cured	Safe sex advice and assessment of re-infection not described
Pareek and Chowdhury (1981) [35]	Non-randomised, comparator study	Men with urethral gonorrhoea infection (culture positive and beta lactamase detected), Riyadh, Saudi Arabia	Single-dose gentamicin 160 mg intramuscular injection (*n* = 20)	Single-dose spectinomycin 2 g intramuscular injection (*n* = 20)	Cure	Safe sex advice, definition and assessment of re-infection not described
					Patients in whom culture on days 3, 7 and 14 post treatment were negative were considered cured	
Yoon et al. (1988) [34]	RCT (random numbered tickets used to divide patients into 2 groups)	Men with uncomplicated gonococcal urethritis (Gram stain and ‘bacteriological test of urethral secretions’), Seoul, Korea	Single-dose gentamicin 240 mg intramuscular injection (*n* = 137)	Single-dose kanamycin 2 g intramuscular injection (*n* = 137)	Cure	All patients advised to avoid sexual intercourse during the period of treatment. Definition and assessment of re-infection not described
					Cases with negative Gram stain and bacteriological test (undefined) of urethral secretions	
Lule et al. (1994) [28]	RCT (computerised randomisation)	Men presenting with urethral discharge +/-dysuria and gram-negative intracellular diplococci on urethral smear and/or positive culture, Malawi	Single-dose gentamicin 240 mg intramuscular injection (*n* = 40)	Amoxicillin 3 gm, probenecid 1 gm, and clavulanate 125 mg by mouth once (*n* = 60)	To determine the relative contribution of gonorrhoea and chlamydia to urethritis in Malawi	Safe sex advice not described
				Amoxicillin 3 gm, probenecid 1 gm, and clavulanate 125 mg, by mouth once and doxycycline 100 mg BD for 7 days (*n* = 56)	To evaluate the effectiveness of five antibiotic therapies for urethritis	6/48 (12.5%) patients with persistent gonococcal infection at follow-up reported having sex between initial and follow-up visits compared to 21 of 249 (8.4%) men for whom gonococcal infection was not detected at follow-up (*p* = 0.4)
				Ciprofloxacin 250 mg by mouth once (*n* = 59)	Cure not defined. An assessment of symptoms and signs, urethral Gram stain and culture were obtained at 8–10 days post treatment	
				Co-trimoxazole (trimethoprim 320 mg/sulphamethoxazole 1,600 mg) by mouth for 2 days (*n* = 29)		

*RCT* randomised control trial, *mg* milligrams, *g* grams, *BD* twice daily.

### Assessment of risk of bias

The included studies were assessed for risk of bias using either the Cochrane risk of bias tool (randomised studies) or the GRACE checklist (observational studies). Study methodology was poorly reported, so that consistently assessing methodological quality and risk of bias in each individual study was difficult. Insufficient detail was often included in the publication to distinguish accurately what was done in contrast to what was reported. As such, risk of bias within and across individual studies was unclear or judged by reviewers to be high (Additional file 2).

### Effectiveness of gentamicin

**Figure 2:**
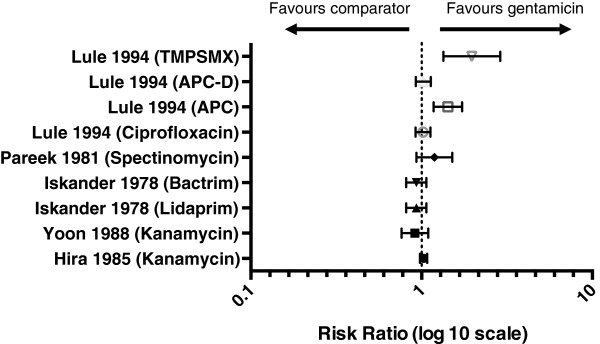
**Efficacy of gentamicin.** The probability of cure following treatment with gentamicin compared to cure with a comparator antibiotic. The probability of cure was comparable between gentamicin and comparator antibiotics. *TMPSMX* trimethoprim/sulphamethoxazole, *APC* amoxicillin, probenecid and clavunate, *APC-D* amoxicillin, probenecid, clavunate and doxycycline. Risk ratios: Lule (TMPSMX) 1.9679 (95% CI 1.3412-2.8873); Lule (APC-D) 1.0231 (95%CI 0.924201.1325); Lule (APC) 1.4250 (95% CI 1.1754-1.7275); Lule (ciprofloxacin) 1.0191 (95% CI 0.9231-1.1251); Pareek (specinomycin) 1.1875 (95% CI 0.9331-1.5113); Iskander (Bactrim) 0.9310 (95% CI 0.8122-1.0672); Iskander (Lidaprim) 0.9310 (95% CI 0.8122-1.0672); Yoon (kanamycin) 0.9142 (95% CI 0.7630-1.0954); Hira (kanamycin) 1.0280 (95% CI 0.979301.0791).

All five studies reported cure, as defined in Table 1, as a primary outcome (Table 2). Fixed effects meta-analysis was not performed for any of the defined outcomes due to the high level of clinical heterogeneity between studies and the high risk of bias within individual studies (Additional file 2). Whilst cure, presented as a percentage rate, was reported in each of the included studies, they differed significantly in definition of cure (clinical cure or negative microscopy and/or culture), timing of cure and their assessment of re-infection (Additional file 3).Figure 2 summarises the efficacy of gentamicin in the treatment of uncomplicated gonorrhoea infection. The probability of cure was comparable between gentamicin and comparator antibiotics.

**Table 2:** Outcome data of included studies

**Outcome**	**Hira et al. **[36]	**Iskandar et al. **[33]	**Pareek and Chowdhury **[35]	**Yoon et al. **[34]	**Lule et al. **[28]
**Cure**	Gentamicin: 98% (216/220)	Gentamicin: 27/30 (90%)	Gentamicin: 19/20 (95%)	Gentamicin: 78/125 (62.4%)	Gentamicin: 38/40 (95%)
	Kanamycin: 95% (85/89)	Co-trimoxazole: 29/30 (96.6%)	Spectinomycin: 16/20 (80%)	Kanamycin: 86/126 (68.3%)	Ciprofloxacin: 55/59 (93%)
		Lidaprim: 29/30 (96.6%)		23 patients did not attend follow-up and were excluded	APC: 40/60 (67%)
		Adjusted to include only those attending on day 7:			APC-D: 52/56 (93%)
		Gentamicin: 19/22 (86.4%)			Co-trimoxazole: 14/29 (48%)
		Bactrim: 15/16 (93.7%)			
		Lidaprim: 20/21(95.2%)			
**Need for additional treatment**	No data	No data	No data	No data	No data
**Adverse event**	‘No serious toxicity or other adverse reactions were noticed in either group of men. Serum creatinine values were normal in the 52 patients given gentamicin and the 28 kanamycin whose blood samples were tested’	‘No adverse side effects were observed in any of the patients’	‘There were no obvious side effects with either of these drugs. The blood urea and creatinine values remained within normal limits’	‘There was no side effect of using kanamycin and gentamicin’	No data
**Hospital attendance**	No data	No data	No data	No data	No data

*APC* amoxicillin, probenecid and clavulanate, *APC*-*D* amoxicillin, probenecid, clavulanate and doxycycline, *TMPSMX* trimethoprim/sulphamethoxazole.

## Discussion

Our systematic review found insufficient data to support or refute the role of gentamicin in the treatment of gonorrhoea infections. Five studies of single-dose intramuscular gentamicin for the treatment of uncomplicated gonococcal urethritis in men met inclusion criteria and reported cure rates of 62% to 98%. The probability of cure was comparable between gentamicin and comparator antibiotics.

A separate systematic review assessing the effectiveness of gentamicin for uncomplicated urogenital gonorrhoea infection has recently been published [37] and reported a pooled percentage with negative culture after single-dose gentamicin of 91.5% (95% CI 88% to 94%). It included three studies of which only two met our inclusion criteria due to methodological differences [36]. Firstly, Dowell and Kirkcaldy included studies with historical controls and single-arm case series. Secondly, they included studies in which gentamicin was given as part of a treatment regimen in combination with other antibiotics. Thirdly, their definition of gonorrhoea was limited to participants with uncomplicated urogenital infection and diagnosis was restricted to urethral or cervical culture at the time of treatment and follow-up.

Our data supports the conclusion that gentamicin may not achieve the 95% cure rate recommended by the World Health Organization for empirical therapy. However, the risk of bias within available studies limits any firm conclusions being drawn and a potential role for gentamicin as an alternative or adjunctive agent remains and merits evaluation in randomised trials. Preliminary data from an American study examining the effectiveness of gentamicin with azithromycin recently suggested high efficacy (202/202 negative culture at 10–17 days post treatment) but poor tolerability of this regimen (27.7% reporting nausea and 47% any gastrointestinal disturbance) [38]. Also, this study did not determine the efficacy of the individual antibiotics, or efficacy of gentamicin for extra-genital infections, and further randomised control trials incorporating currently recommended antibiotic regimens, comparing different gentamicin doses and correlating *in vitro* gentamicin susceptibilities to clinical response are needed.

A comprehensive review of the literature was performed with all relevant identified articles obtained and translated. Few studies met the inclusion criteria with limited numbers of patients receiving single-dose intramuscular gentamicin. The five studies included for data extraction were performed in Malawi, Zambia, Egypt, Korea and Saudi Arabia, and their findings may not be applicable to other settings where first-line treatment regimens and gentamicin susceptibility may differ. No studies of genital infection in women or non-genital gonorrhoea infection were identified by the search strategy, and the findings cannot be extrapolated to these groups.

### Quality of included studies

Two of the identified studies were very small with 20 [35] and 30 [33] patients receiving gentamicin. The comparator antibiotic varied across the included studies, and all were suboptimal when compared to current UK management guidelines. Two studies were described as randomised control trials, but none adequately described their method of generating a random allocation sequence, method of concealment or blinding to the allocation schedule. In addition, differences in definition of gonococcal infection, gentamicin dosing, comparator antibiotic, evaluation of re-infection and definition and timing of cure meant that it was inappropriate to pool data for meta-analysis.

## Conclusions

Based on current evidence, there are insufficient data to support or reject a recommendation for inclusion of single-dose gentamicin as a first-line agent in the treatment of uncomplicated gonorrhoea infection. Further high-quality RCTs incorporating currently recommended antibiotic regimens with laboratory measurement of gentamicin MIC are needed to inform a change in clinical practice.

## Competing interests

The authors declare that they have no competing interests.

## Authors’ contributions

EH and DD conducted the review and writing of the manuscript. LD reviewed the protocol, gave statistical advice and reviewed the manuscript. JDCR contributed to the initial concept and reviewed and revised the manuscript. All authors read and approved the final manuscript.

## Supplementary Material

Additional file 1 **Search strategy.** Search strategy used to identify studies for inclusion in the review. Click here for file

Additional file 2 **Risk of bias assessment.** Summary of the risk of bias in each included study. Click here for file

Additional file 3 **PRISMA statement.** Checklist against PRISMA guidelines for the reporting of systematic reviews. Click here for file
