# Supplementary material for: The effectiveness of gentamicin in the treatment of Neisseria gonorrhoeae: a systematic review
Source: Syst Rev. 2014 Sep 19;3:104. doi: 10.1186/2046-4053-3-104 (PMC4188483; doi:10.1186/2046-4053-3-104)
Supplement: Additional file 1 — Search strategy. Search strategy used to identify studies for inclusion in the review. [file 2046-4053-3-104-S1.docx]

**Additional file 1: Search strategy**

**Healthcare Databases Advanced Search:** [**http://www.library.nhs.uk/hdas**](http://www.library.nhs.uk/hdas)

**Medline**

1. MEDLINE; exp GONORRHEA/

2. MEDLINE; exp NEISSERIA GONORRHOEAE/

3. MEDLINE; gonorrhoea.ti,ab

4. MEDLINE; gonorrhea.ti,ab

5. MEDLINE; 1 OR 2 OR 3 OR 4

6. MEDLINE; exp GENTAMICINS/

7. MEDLINE; gentamicin.ti,ab

8. MEDLINE; 6 OR 7

9. MEDLINE; 5 AND 8

**Cinahl**

10. CINAHL; exp GONORRHEA/

11. CINAHL; exp NEISSERIA GONORRHOEAE/

12. CINAHL; gonorrhoea.ti,ab

13. CINAHL; gonorrhea.ti.ab

14. CINAHL; 10 OR 11 OR 12 OR 13

15. CINAHL; exp GENTAMICINS/

16. CINAHL; gentamicin.ti,ab

17. CINAHL; 15 OR 16

18. CINAHL; 14 AND 17

**Embase**

19. EMBASE; exp GONORRHEA

20. EMBASE; exp NEISSERIA GONORRHOEAE/

21. EMBASE; gonorrhoea.ti,ab

22. EMBASE; gonorrhea.ti.ab

23. EMBASE; 19 OR 20 OR 21 OR 22

23. EMBASE; exp GENTAMICINS/

24. EMBASE; gentamicin.ti,ab

25. EMBASE; 23 OR 24

26. EMBASE; 23 AND 26

**Cochrane Library: www.thecochranelibrary.com/**

Gonorrhea or Neisseria Gonorrhoeae or Gonorrhoea or Gonorrhea and Gentamicin

**NICE:** [**www.nice.org.uk/**](http://www.nice.org.uk/)

Gonorrhea or Neisseria Gonorrhoeae or Gonorrhoea or Gonorrhea and Gentamicin

**SIGN:** [**www.sign.ac.uk/**](http://www.sign.ac.uk/)

Gonorrhea or Neisseria Gonorrhoeae or Gonorrhoea or Gonorrhea and Gentamicin

**Clinical trials.gov:** [**www.clinicaltrials.gov**](http://www.clinicaltrials.gov)

**WHO International Clinical Trials Registry Platform:** [**www.who.int/trialsearch**](http://www.who.int/trialsearch)

**Google Scholar:** [**www.scholar.google.com/**](http://www.scholar.google.com/)

Gonorrhea or Neisseria Gonorrhoeae or Gonorrhoea or Gonorrhea and Gentamicin

**Grey literature search**

Aggressive Research Intelligence Facility (ARIF), Birmingham University

British Library

Clinical Trials.gov

Cochrane Central Register of Controlled Trials

Current Controlled Trials

Google Scholar

Open Grey

UK Clinical Research Network
