# Supplementary material for: The effectiveness of gentamicin in the treatment of Neisseria gonorrhoeae: a systematic review
Source: Syst Rev. 2014 Sep 19;3:104. doi: 10.1186/2046-4053-3-104 (PMC4188483; doi:10.1186/2046-4053-3-104)
Supplement: Additional file 2 — Risk of bias assessment. Summary of the risk of bias in each included study. [file 2046-4053-3-104-S2.docx]

**Additional file 2: Summary of assessment of bias**

Risk of bias in randomised studies was assessed using the risk of bias tool described in the Cochrane Handbook for Systematic Reviews of Interventions [31]. A score was initially allocated to each of the six domains in the risk of bias tool for each individual study. A summary of the risk of bias assessment across included studies is provided below.

|  | **Adequate sequence generation** | **Allocation concealment** | **Blinding** | **Incomplete outcome data addressed** | **Free of selective reporting** | **Free of other bias** |
| --- | --- | --- | --- | --- | --- | --- |
| **Lule [28]** | **+** | **+** | **?** | **-** | **?** | **?** |
| **Hira [31]** | **-** | **?** | **?** | **+** | **+** | **?** |
| **Iskandar [32]** | **?** | **?** | **-** | **?** | **?** | **?** |
| **Yoon [33]** | **-** | **?** | **?** | **-** | **-** | **?** |

**KEY:**

- High risk of bias

+ Low risk of bias

? Unclear risk of bias

The GRACE checklist [32] was used to rate the quality of the non-randomised observational study [36] included in the review. This checklist does not have a scoring system and a summary of the assessment is provided below

| **DATA** |  |
| --- | --- |
| D1. Were treatment and/or important details of treatment exposure adequately recorded for the study purpose in the data sources? | (+) Yes, reasonably necessary information to determine treatment or intervention was adequately recorded for study purposes |
| D2. Were the primary outcomes adequately recorded for the study purpose? | (-) No, data source clearly deficient |
| D3. Was the primary *clinical* outcome measured objectively rather than subject to clinical judgment? | (+) Yes, clinical outcome was measured objectively |
| D4. Were primary outcomes validated, adjudicated, or otherwise known to be valid in a similar population? | (-) No, *or* not enough information in article. |
| D5. Was the primary outcome measured or identified in an equivalent manner between the treatment/intervention group and the comparison groups? | (+) Yes. |
| D6. Were important covariates that may be known confounders or effect modifiers available and recorded? Important covariates depend on the treatment and/or outcome of interest | (+) Yes, most if not all important known confounders and effect modifiers available and recorded. |
| METHODS |  |
| M1. Was the study (or analysis) population restricted to new initiators of treatment or those starting a new course of treatment? | (+) Yes, only new initiators of the treatment of interest were included in the cohort. |
| M2. If 1 or more comparison groups were used, were they concurrent comparators? If not, did the authors justify the use of historical comparison groups? | (+) Yes, data were collected during the same time period as the treatment group (“concurrent”). |
| M3. Were important confounding and effect modifying variables taken into account in the design and/or analysis? | (-) No, some important covariates were available for analysis but not analyzed appropriately. |
| M4. Is the classification of exposed and unexposed person-time free of “immortal time bias”? | (+) Yes. |
| M5. Were any meaningful analyses conducted to test key assumptions on which primary results are based? | (-) Not enough information in article. |
